# Self-based goals, underlying reasons, performance and discrete emotions among parkrunners

**DOI:** 10.3389/fpsyg.2023.1017836

**Published:** 2023-07-03

**Authors:** Mairi Mulvenna, James W. Adie, Carlo Tramontano

**Affiliations:** ^1^Faculty of Arts, Science and Technology, University of Northampton, Northampton, United Kingdom; ^2^Coventry University, Coventry, United Kingdom

**Keywords:** self-based goals, self-determination, stress, performance, discrete emotions

## Abstract

**Introduction:**

A temporal sequence of hypothesized relationships was tested between self-based goals and their underlying reasons → stress appraisals → performance and emotions, among UK parkrunners. A conditional process model was also examined to ascertain the potential moderating role of self-determined reasons in explaining the indirect relationship of self-based goals predicting performance and emotions via stress appraisals.

**Methods:**

Utilizing a prospective design, 324 parkrunners (*M*_age_ = 45.27; SD = 10.73 years) completed online measures of self-based goals, their underlying reasons at 7 days (T1), and stress appraisals at 24 h (T2), prior to their next UK parkrun. Performance data and discrete emotions (pride and shame) were reported 24 h post-parkrun (T3).

**Results:**

Structural Equation Modeling revealed partial support for the hypothesized model. More specifically, findings suggested that: (1) T1 self-determined reasons underpinning a self-approach goal positively predicted T2 challenge appraisals and T3 pride, (2) T1 self-determined reasons for pursuing a self-avoidance goal corresponded to reduced T3 performance and shame, (3) T2 challenge and threat appraisals were found to positively relate to T3 pride, and (4) the slower parkrunners ran, the more shame they felt post-event. T2 challenge and threat appraisals were found to mediate the relationship between T1 self-determined reasons underlying a self-approach goal and T3 pride. Further analysis failed to support a conditional process model.

**Discussion:**

Our findings suggest the intensity of pursuing a self-based goal does not matter at all, but underlying self-determined reasons are a key driver influencing stress appraisals, performance and subsequent emotions among parkrunners.

## Introduction

Participation in organized sport has the potential to elicit adaptive achievement-related cognitions, emotions, and behaviors (Fraser-Thomas and Côté, 2009; Adie and Bartholomew, 2013). However, it is important to note that mere participation alone does not automatically guarantee such outcomes, and for some, maladaptive consequences may ensue (Quested et al., 2013). For example, athletes frequently exhibit feelings of extreme pressure from the sporting demands they encounter, sub-standard performances, negative emotions along with intentions of, and actual, sport drop-out (e.g., Krane et al., 1997; Fraser-Thomas and Côté, 2009).

From a motivational perspective, achievement goal approaches (AGA; Nicholls, 1984; Dweck, 1986; Elliot, 1999; Elliot et al., 2011) have extensively contributed to our knowledge in explaining variability of the psychological and emotional functioning of sport performers (e.g., Kavussanu et al., 2015). Recent advancements in the achievement motivation literature (e.g., Vansteenkiste et al., 2014a,b; Delrue et al., 2016) have further demonstrated an enhanced understanding in predicting sport-related outcomes by considering the ‘why’ underpinning achievement goal adoption. In the current study, we drew from an integrated theoretical perspective (Vansteenkiste et al., 2014a,b) by examining the hypothesized relations between self-based goal pursuits (adopted from the 3 × 2 Achievement Goal Model [AGM]; Elliot et al., 2011) and their underlying reasons (adopted from Self-Determination Theory [SDT], Deci and Ryan, 1985) on stress appraisals, subsequent performance and discrete emotions, over time, among recreational runners. We also explored the possible moderating role of the reasons underlying self-based goal pursuit on these outcomes, as well as the potential mediating effects of stress appraisals.

### Achievement goal approach

The AGA (e.g., Dweck, 1986; Elliot, 1999; Elliot et al., 2011) has permitted the study of competence-based pursuits in achievement settings for over the past 30 years. Early theorists (e.g., Nicholls, 1984; Dweck, 1986) distinguished between mastery (i.e., focused on self-improvement and task mastery) and performance-based goals (i.e., focused on outperforming others), commonly referred to as the dichotomous goal framework. More recently, the hierarchical model of achievement motivation (HMAM; e.g., trichotomous goal framework, Elliot and Harackiewicz, 1996; the 2 × 2 achievement goal model, McGregor and Elliot, 2002) was developed by reconceptualizing mastery and performance goals into separate approach and avoidance constructs. In the sport-based literature, studies have repeatedly shown mastery-approach goals to be associated with adaptive achievement patterns, enhanced performance and increased well-being (see Harwood et al., 2008; Adie and Bartholomew, 2013; Lochbaum and Gottardy, 2015 for reviews). However, the validity of these findings can be drawn into question when considering mastery-based goals have been operationalized across two different standards of evaluation, namely task-based competence (i.e., doing well or not doing poorly in relation to the demands of a task) and self-based competence (i.e., doing well or not doing poorly in relation to previous performance), yet studies have mostly employed measures to assess mastery approach and avoidance goals which only capture one standard (see Mascaret et al., 2015).

In addressing these limitations, the latest version of the HMAM, the 3 × 2 achievement goal model (3 × 2 AGM; Elliot et al., 2011), proposed and found four different mastery-based goals to be salient in education (e.g., Elliot et al., 2011) and sport (Mascaret et al., 2015; Wang et al., 2017) settings: (1) task-approach (TAp) goals refer to striving to attain task-based competence, (2) task-avoidance (TAv) goals relate to the avoidance of task-based incompetence, (3) self-approach (SAp) goals reflect striving to attain self-based competence, and (4) self-avoidance (SAv) goals represent a focuses to avoid self-based incompetence. The former performance-based goals were now known as an other-approach (OAp; involves aiming to outperform others) and other-avoidance (OAv; focuses on avoiding performing any worse relative to others) goal. To extend on these definitions, in the full model, self-based goals are explicitly conceptualized in terms of both an individual’s past and potential accomplishments, but, when measured via questionnaire items, they are referred to solely in terms of the past (e.g., SAp: ‘I aim to do well in this race relative to how well I have done in the past on this course’; SAv: ‘I am to avoid running any worse in this race than I have done on previous attempts on this course’). Some work has started to focus exclusively on self-based goals that encompass one’s potential (i.e., potential-based goals) in the education environment (Elliot et al., 2015), however, overall, the study of SAp and SAv goals is still very much in its infancy. Most research that has examined self-based goals has done so in educational settings (e.g., Elliot et al., 2011, 2015; Benita et al., 2017) with less attention received in the sport domain (e.g., Mascaret et al., 2015; Lower and Turner, 2016; Wang et al., 2017). Nevertheless, there is a strong argument that self-based goals are widely endorsed by participants across different sports (e.g., Wang et al., 2017) and levels of participation (Lower and Turner, 2016). For the purpose of the current study, we were only interested in understanding the implications of pursuing self-based goals (in relation to past performances) in a sport setting.

In addressing the dearth of sport research on self-based goals, we examined SAp and SAv goals among parkrunners. With almost 150,000 events and over 2 million registered runners in the UK alone, parkrun predominantly attracts novice to elite runners to take part in a weekly, timed, 5K event. For parkrunners, improving upon previous performances and striving to achieve personal best (PB) times appear to be of great importance (Stevinson et al., 2015; Sharman and Nash, 2018; Tulle et al., 2018) with over five million PBs recorded since its creation in 2004. Empirical research has further supported the notion that participants engaging with distance limited events (i.e., running events ranging from 5 km to ultramarathons where the aim of completing is to do so in the shortest possible time) hold a drive to improve upon their previous performances. In research focusing on female ultrarunners, it was reported a primary goal pursuit for participants revolved around personal achievement, more specifically finishing a distance within a given time (Krouse et al., 2011). For some women, a time goal meant making the cut-off time to complete the race, and for others, it was about completing a previously raced course in a faster time. Later research conducted among half-, full-, and ultra-marathon runners across genders and different ability groups (i.e., recreational vs. serious; Kruger and Saayman, 2013; Hanson et al., 2015), replicated this pattern of findings as PB strivings were at the forefront of all achievement pursuits. Furthermore, Delrue et al. (2016) explored achievement pursuit with competitive runners (taking part in a 20 km event). Utilizing the dominant goal method for assessing achievement pursuits (Van Yperen, 2006), researchers reported the most important goal for athletes participating in a running event was the SAp goal, closely followed by a SAv goal. Interestingly, across all of these studies, competence-based pursuits reflecting performance goals (i.e., attempts to outperform, or avoid doing any worse than, your opponents) were rated amongst some of the lowest influencing motivational factors. This further alludes to the salience of self-based goals in sport and running in particular; individual’s goal endorsements place greater focus on doing well or not doing worse compared to one’s previous performance (Roebuck et al., 2018). We would expect this would be no different among those engaging with parkrun – after all, evidence exists that self-referenced goals represent a key achievement striving within this population (Stevinson et al., 2015; Sharman and Nash, 2018). One shortcoming of this literature surrounds the absence of a theoretical framework in which to study goal pursuits. Although past researchers have focused on constructs (e.g., PB goals; see also Martin, 2006) similar to the self-based goal (particularly the SAp), they were not exclusively operationalized in this way. To advance the extant achievement goal literature in sport, we studied the implications of adopting SAp and SAv goal, as conceptualized by the 3 × 2 AGM (Elliot et al., 2011), on performance and achievement emotions among parkrunners.

Few studies have examined the link between self-based goals and performance in achievement settings. One exception that has examined the implications of self-based goals on running performance using the 3 × 2 AGM (Elliot et al., 2011) is a study by Delrue et al. (2016). These authors reported a significant and positive association between SAp goals and aspired time, and also SAp goals and faster race time in relation to individuals in pursuit of a SAv goal among a sample of long-distance runners. A primary limitation of this work, however, relates to how researchers implemented their goal measurement. By incorporating the dominant achievement goal method (Van Yperen, 2006), Delrue et al. (2016) did not directly assess participants’ endorsement of self-based goals, rather they were ranked relative to the endorsement of other-based goals. More literature exists within education, However, an inconsistent pattern of findings between SAv goals and achievement/performance outcomes has emerged here. For example, David (2014) showed that SAv goals negatively related to test performance whilst Luftenegger et al. (2016) directly contrasted this, revealing significant and positive correlation between SAv goals and achievement. Moreover, Gillet et al. (2017) found no relation between both SAp or SAv goals and achievement (indexed by passing or failing the semester) among their sample. These equivocal findings may be explained through the cultural differences in population samples tested, distinct educational subjects explored, and the various indicators employed to assess achievement/performance. Another factor that may play a part in influencing the varied findings, is the research design. In the current study we examined the temporal association between self-based goals and parkrunners’ performance (operationalized by 5 km running time). Well-selected measurement intervals as depicted in a temporal research design are also essential in mediation analysis (an objective of this study). Gathering longitudinal data permits the researcher to demonstrate that the causal variable has sufficient time to influence the mediator, which in turn has sufficient time to influence the outcome (Cole and Maxwell, 2003; Collins, Graham, and Flaherty, 1998). Moreover, it is important to consider that a key source of self-based competence representing a striving to do better or worse than a past ‘running’ performance, for this group, is time (serving as an indicator of performance success/failure).

### Achievement goals, emotional functioning and performance

An additional objective of the current study was to examine how self-based goals would be associated with post-performance emotions. This represents a gap in the literature, as the majority of sport research has focused on emotions that predict performance (e.g., pre-competitive anxiety). In the past, many studies have measured emotions by taking a broader ‘affect’ approach (i.e., positive and negative affect). Such an approach has been criticized (e.g., Jones et al., 2005). It obscures insightful information, and in the current context, it could limit understanding of how the potential relationships between achievement goals and specific emotions could unfold in achievement contexts. In short, emotions and affect are two different concepts. Emotions are defined as “relatively brief but intense experiences activated by cognitive appraisal of a situation” (Lane and Terry, 2000, p. 17), whereas affect is a “broad rubric that refers to all things emotional” (Rosenberg, 1998, p. 247). To extend on this, affect represents quick and simple evaluation of something as good or bad, pleasurable or contributing to a feeling of displeasure (Jekauc et al., 2021). Emotions on the other hand, represent a progression on affect that typically involves physiological arousal, emotion expression, and obvious higher cognitive processing (Jekauc et al., 2021). Further, these variables differ in that each emotion has a specific associated antecedent (Lazarus, 1991, 2000), as opposed to affect which has no explicit referent. Measuring specific emotions may be superior to assessing a composite score of affect because this can capture the variations in specific emotional experiences of competing individuals (Jones et al., 2005).

Pekrun (1992) and Pekrun et al. (2002, 2006) developed a taxonomy of emotions. Pertinent to the sporting environment are achievement emotions, defined as “emotions that are directly linked to achievement activities or achievement outcomes” (Pekrun et al., 2011, p. 37). Pekrun (1992) identified two dimensions of particular importance for achievement emotions as object focus and valence (positive vs. negative emotions). Object focus categorizes emotions as either (1) activity-related, (e.g., enjoyment of learning) or (2) outcome-related (to be ether prospective or retrospective [e.g., hope for success or shame following failure]). Sport research that has generally investigated the presence of emotions in athletes has indeed revealed a wide-ranging spectrum of experiences (e.g., Nicholls et al., 2010; Martinent et al., 2012), however, little exists with respect to the emotional experiences post-event. For the purposes of this study, we exclusively focused on outcome related emotions.

Although the achievement goal and affect (as an indicator of subjective well-being; see Diener, 1984) sport literature is relatively well-established, there is less work studying the link between achievement goal pursuit and achievement emotions (also called discrete emotions). A notable exception is the work conducted by Dewar and Kavussanu (2012). Their work found athletes in pursuit of a task-based (i.e., mastery) goal were more likely to experience happiness, pride, and hope (and less dejection and shame) post-performance relative to those following an other-based goal within a competitive team sport environment. Later experimental work (Dewar et al., 2013) found the ego-orientated (i.e., performance/other-based goal) group to experience greater pre-competition excitement and anxiety than the task-oriented group on an agility task. Other work in the physical domain (e.g., Mouratidis et al., 2009; Lochbaum and Stevenson, 2014) revealed PE students who were task involved (or in pursuit of a mastery-approach goal) predicted positive activating emotions (e.g., pride, hope and enjoyment) whilst being inversely related to negatively valanced emotions (i.e., anxiety, anger, shame, hopelessness and boredom). Ego-involved participants (or those pursuing an other-based goal) exhibited a mixed picture as they were positively associated with pride and all the negative emotions, a pattern central to the debate surrounding the utility of these goals in the literature (see Senko et al., 2011).

Although these studies provide encouraging findings for the achievement motivation and emotion literature within the physical domain, from a conceptual viewpoint, they are embedded within early motivation theories (i.e., the dichotomous and trichotomous frameworks), focusing on motivational climates, rather than specific achievement goal pursuit. In that respect, researchers have not yet explicitly tested the goal constructs of the most recent 3 × 2 AGM (Elliot et al., 2011), specifically self-based goals, which remain under-researched. Therefore, conclusions cannot be drawn on whether a similar pattern of findings would remain for these constructs, which we focus on in the present research. Moreover, the limited work existing exploring emotional experiences has focused on athletes’ goals operating within team sports (e.g., Dewar and Kavussanu, 2012), so less is known about how individual sport participants function as a result of their self-based goal pursuits in competition.

To fill current voids in the literature and in an attempt to provide a greater, consistent understanding of the motivational processes in sport, our first aim was to understand to what degree participants pursuing SAp and SAv goals could contribute to the performance and emotional experiences among parkrunners. We chose to exclusively focus on parkrunners experiences of pride and shame as retrospective emotions, when reflecting on how they felt post-event about their performance. Pride is defined as a feeling or deep pleasure or satisfaction derived from one’s own achievement whilst shame as the direct opposing emotion can be described as a feeling of humiliation or distress caused by the consciousness of failure (Pekrun, 1992). When reflecting on previous literature that highlights the relevance of personal achievement and satisfaction for participants involved within the running community, it was expected these two emotions would be highly salient among our parkrunners (e.g., Krouse et al., 2011; Roebuck et al., 2018). *Our first hypothesis (H^1a^) therefore, was that individuals in pursuit of a SAp goal, would run a faster time, as well as experiencing greater feelings of pride and less shame post-parkrun. Due to the known detrimental effects associated with avoidance goals in sport* (see Papaioannou et al., 2012)*, we tentatively expected those in pursuit of a SAv goal to experience less pride and more shame post-parkrun (H^1b^).*

### Self-determination theory

A complimentary motivational framework that has the potential to enhance the predictive utility of self-based goals is SDT (Deci and Ryan, 1985). One of the central tenets of this theory is that (goal-directed) behavior is regulated by either autonomous or controlling motivation (i.e., in the current study, the reasons underpinning an individual’s self-based goal strivings). Autonomous motivation refers to behaving with free volition, engaging with an activity because of the interest, fun and challenge it provides. In contrast, controlling forms of regulation represent behavior that is performed to avoid feelings of personal guilt and shame, or because of external contingencies (e.g., for a reward or to avoid punishment). Based on theoretical propositions, it is assumed autonomous regulation will lead to a more adaptive and optimal form of athlete functioning, whilst controlling regulation is expected to result in diminished functioning. The majority of research in this field has consistently found autonomous forms of regulation to be associated with higher adaptive consequences, such as greater persistence, more positive affect, enhanced performance, and well-being (Deci and Ryan, 2008). Controlled regulation on the other hand, has consistently been linked with detrimental outcomes, such as increased ill-being, negative affect and poor task performance (for a review, see Deci and Ryan, 2000).

In alignment with other SDT researchers (e.g., Spray and Wang, 2001; Levesque et al., 2004; Ommundsen and Kvalo, 2007; Ciani et al., 2011) we created a relative autonomy index (RAI) to reflect our second study aim that was to examine whether more or less self-determined reasons underpinning goal pursuits would influence parkrunners’ performance (indexed by 5 km finishing time) and discrete emotions. *Our second set of hypotheses expected that more self-determined reasons underlying SAp goals would be linked to increased performance (i.e., running a faster parkrun time) and pride along with reduced levels of shame (H^2a^). We also predicted that less self-determined reasons for adopting a SAv goal would lead to reduced performance (i.e., reflected by a slower parkrun time) and pride, and increased levels of shame (H^2b^).*

### Goals, underpinning reasons, well-being and performance

According to Elliot’s perspective of the Elliot and Conroy, 2005 it was proposed there were varying reasons underpinning goal pursuit, and these reasons may not only activate goal pursuit but also help shape their consequential effects (Elliot and Thrash, 2001). Therefore, the same goal may function differently based on the underlying reasons for pursuing it. This idea involves disentangling all reasons from the goal, exclusively defining them as aims, and then recombining the aim (i.e., the goal) with each unique reason, a special type of interaction coined “goal complexes” in the achievement goal literature (Senko and Tropiano, 2016). Based upon this reconceptualization, researchers have been presented with an opportunity to more rigorously address the regulation of achievement goals, investigating potential different types of reasons underlying any one goal, rather than isolating and comparing the two elements (Senko and Tropiano, 2016). However, the notion of goal complexes remains under-researched within AGA and sport-based research, with existing work focusing mostly on goal antecedents (activation), not how the regulation of goals or combined goal complexes influence outcomes. In order to extend this line of Inquiry, this study will assess the unique and moderating effects of underlying reasons and self-based goals on performance and emotions.

In terms of the moderation hypothesis, which also forms part of Vansteenkiste et al.’s (2014a) framework, seldom studies have explicitly tested this assumption in the achievement goal literature. One recent study aiming to address this gap, albeit in education, revealed the relationship between OAp goals and goal attainment to be moderated by autonomous goal motivation (Gillet et al., 2014). Precisely, OAp goals were more strongly related to higher goal attainment for students with greater compared to lower autonomous goal motivation, however, these findings were not replicated in their follow-up studies within work settings (Gillet et al., 2014), leaving evidence scant and inconsistent.

Previous sport studies have attempted to integrate AGA’s (e.g., Elliot and McGregor, 2001), with SDT (Deci and Ryan, 1985) toward predicting emotions and affect and performance in sport. Among the first to explore this goal-complex notion using amateur soccer players, was a study by Vansteenkiste et al. (2010a). The authors reported autonomous reasons underlying other-approach (OAp) goals to be positively associated with well-being (e.g., subjective vitality and positive affect) whereas underlying controlling reasons yielded a positive relationship with negative and undesirable outcomes such as immoral functioning (aggressive play). This approach has been further expanded in sport (e.g., Vansteenkiste et al., 2014a,b) and other achievement contexts such as education (e.g., Michou et al., 2014).

Early work, although informative, was conducted in the absence of a guiding theoretical framework. Acknowledging this limitation, Vansteenkiste et al. (2014a) developed a conceptual model for integrating achievement goal theory with SDT. They argued any one goal could lead to somewhat different processes and outcomes, depending on its accompanying reasons, and as such, autonomous and controlled regulations could play a moderating role in the relationship between goals and outcomes. It was proposed these regulations would then relate differently to cognitive, affective, and behavioral outcomes, explaining variance in addition to that accounted for by the strength of the endorsement of achievement goals themselves. For example, SAp goal pursuit for autonomous reasons is likely to be positively associated with adaptive outcomes, however, should the same goal be pursued for controlling reason, it is assumed to be positively related to pressure and less desirable outcomes. A growing body of research, albeit correlational, examined the concomitants of reasons underpinning achievement goal pursuit. For example, Gaudreau and Braaten (2016) concluded that autonomous reasons underlying the OAp and the omnibus mastery-approach goal related to increased positive affect and subjective performance among athletes from various sporting contexts. Controlled reasons of these goals on the other hand were related to less positive and more negative affect. Moreover, the interaction of reasons and achievement goals strengthened the positive association between mastery-approach goals and goal attainment, satisfaction, and positive affect. The above research testing this goal-complex idea, though encouraging, from a conceptual viewpoint is framed within the 2 × 2 AGM (Elliot and McGregor, 2001) whereby the mastery goal remains an omnibus construct, and so which may mask over potential associations between self-based goals only with studied outcomes. Furthermore, researchers focused on affect as an outcome, potentially concealing findings that may result from the interaction of goals and their underlying reasons on achievement emotions. Finally, previous literature has investigated approach-based goals only.

Delrue et al. (2016) did adopt tenets of the 3 × 2 AGM (Elliot et al., 2011) to test the reasons underpinning specific self- and other-based goal constructs in runners. Researchers reported that the reasons component of motivation proved an additional predictive asset next to the goal component. Specifically, researchers reported autonomous reasons underpinning SAp goal pursuit emerged as a positive predictor of aspired time as well as need satisfaction, and actual performance.

Taken together, such findings are consistent with several previous sport studies providing further support for the importance of considering the reasons underlying goal pursuit and the unique role they play in predicting outcomes (e.g., Vansteenkiste et al., 2010a,b; Gaudreau and Braaten, 2016). Despite acknowledging the commonly reported detrimental effects linked with avoidance goals, it warrants further investigation now, when additionally considering the underlying reasons of these goals, if their effects could become less harmful or possibly beneficial (if pursued for autonomous reasons) or even exacerbated if pursued for controlling reasons. Taking all of the above into consideration, the third aim was to test a goal-complex interaction*. Our third set of hypotheses assumed SAp goals would ensure greater adaptive consequences (i.e., increased performance and pride, and reduced shame) when pursued for more self-determined reasons and less benefits if pursued for less autonomous reasons (H^3a^). We also proposed the negative connotations of a SAv goal (i.e., reduced performance and pride, and heightened shame) would be much greater if pursued for less self-determined (i.e., more controlling) reasons, as opposed to autonomous reasons (H^3b^). Finally, it was hypothesized that depending on parkrun time, this could have a positive effect on enhanced feelings of pride (if participants were happy with their performance) or indeed increased levels of shame (if participants were unhappy with their performance; [H^3c^])*.

### The mediational role of cognitive appraisals

Another objective of the study was to understand the psychological mechanisms that may explain the link between self-based goals and their underlying reasons in predicting the 5 km performance and subsequent discrete emotions among a sample of parkrunners. Currently, little is known regarding such mechanisms, but one potential process by which achievement goals might influence athletes’ emotional welfare concerns variability in their cognitive appraisals of stressful events in the sport domain (Adie et al., 2008). According to Lazarus and Folkman’s (1984) Cognitive Appraisal Theory, individual differences exist in cognitively appraising the demands presented in the objective environment and these differences can be categorized as either a challenge or threat. A challenge state is experienced when an individual has sufficient resources available within their environment to meet the perceived demands of a task, viewing the situation as an opportunity for growth or mastery, whereas a threat state occurs when personal resources fail to cope with task requirements, deeming psychological harm potentially imminent. The Theory of Challenge and Threat States in Athletes (TCTSA; Jones et al., 2009) is an alternative framework, extending the work of The Biopsychosocial Model of Challenge and Threat (Blascovich and Tomaka, 1996), that posits key components relevant to the current research, including the acknowledgement that (1) achievement goals determine challenge or threat states in response to competition or sporting activity and (2) both positive and negative emotions can occur whilst individuals are in a challenge state, however, a threat state is linked with negative emotions only. Ultimately, these stress appraisals influence a range of psycho-physiological outcomes and accordingly sport performance (for a full review, please refer to Meijen et al., 2020).

It is assumed and empirically supported in sport settings that achievement goals play a role in determining how an athlete cognitively appraises a potentially stressful performance (e.g., McGregor and Elliot, 2002; Adie et al., 2008; Jones et al., 2009; Adie et al., 2010; Kavussanu et al., 2014; Bartholomew et al., 2017) and also, that cognitive appraisals are relevant to personal well-being and performance (e.g., Giacobbi et al., 2007; Jones et al., 2009). It has been previously demonstrated in empirical research within the running community that autonomous reasons underpinning SAp goal pursuit emerged as a positive predictor of challenge appraisals (Delrue et al., 2016). Further, researchers found controlled reasons undergirding SAp goals yielded somewhat mixed findings with participants appraising the race as both a challenge and a threat. Interestingly, a significant interaction between SAv goal pursuit and controlled motivation in the prediction of pre-race threat appraisals emerged, indicating that runners holding a SAv goal, while standing under pressure, were especially vulnerable to perceive the race as threatening. It appears from this finding that the detrimental effects of avoidance goals are exacerbated when pursued for controlling reasons, at least when appraising an upcoming sporting event.

**Figure 1:**
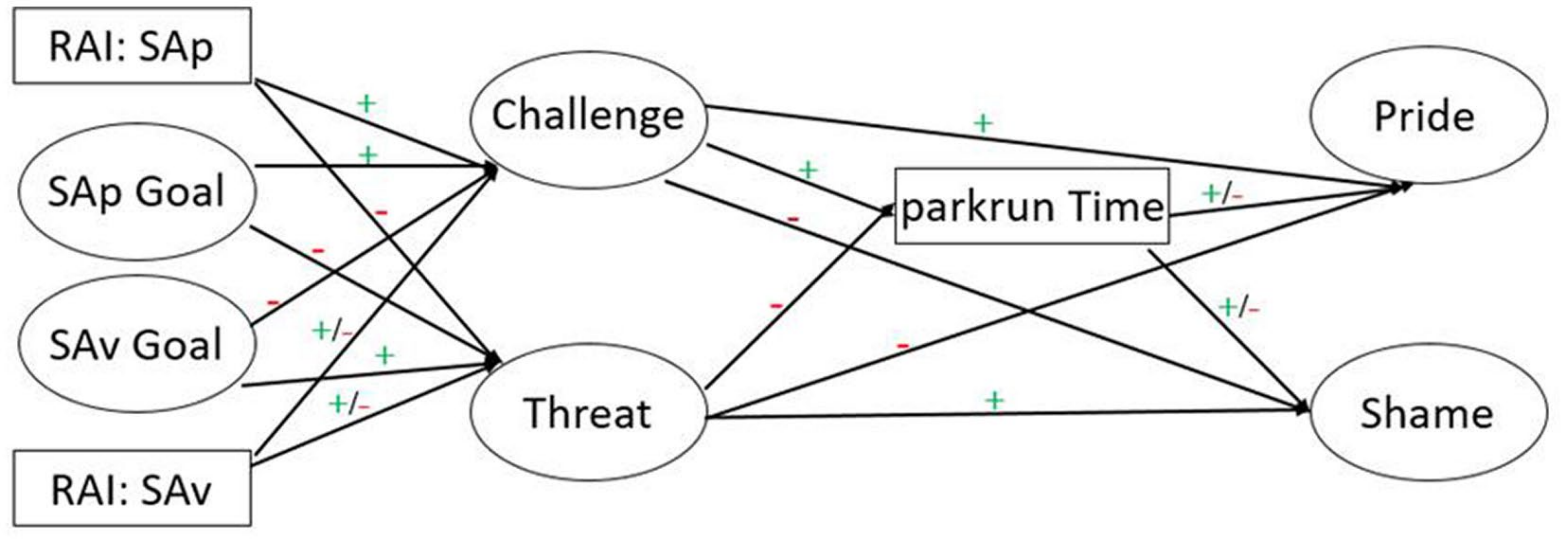
The hypothesized model; expected pathways. SAp = self-approach goal; SAv = self-avoidance goal; RAI_SAp = relative autonomy index underpinning self-approach goals; RAI_SAv = relative autonomy index underpinning self-avoidance goals. Positive direct pathways were also expected between (1) RAI_SAp with parkrun Time and Pride; (2) SAp Goal with parkrun Time and Pride; (3) SAv Goal with Shame; and (4) RAI_SAv with Shame. Negative direct pathways were also expected between (1) RAI_SAp with Shame; (2) SAp Goal with Shame; (3) SAv Goal with parkrun Time and Pride; and (4) RAI_SAv with parkrun Time and Pride.

The temporal research design selected for the present research is also essential in mediation analysis. Gathering longitudinal data permits the researcher to demonstrate that the causal variable(s) have sufficient time to influence the proposed mediators which in turn has sufficient time to influence the outcome (Cole and Maxwell, 2003). The fourth aim of this study was therefore to explore the potential mediating role of stress appraisals between the achievement goal approach and underlying reasons in predicting performance and discrete emotional experience. *Our fourth set of hypotheses expected SAp goals and more self-determined reasons, to be positively associated with challenge appraisals (H^4a^), SAv goals and less self-determined reasons, to be positively related to threat appraisals (H^4b^), challenge appraisals to positively impact performance and experiences of pride, and negatively relate to shame (H^4c^), threat appraisals would demonstrate negative associations with performance and pride, and positive links with increased shame (H^4d^), and appraisals to play a mediating role between goals and/or reasons, with performance and indices of emotional functioning (H^4e^).* To elaborate on H^4e^, positive consequences expected to ensue for SAp goals and/or autonomous reasons via challenge on performance and pride, and detrimental consequences anticipated for SAv goals and/or controlling reasons via threat negatively impacting performance and positively relating to experiences of shame. The hypothesized model is depicted in Figure 1.

### The moderating role of reasons

Extending the work of Delrue et al. (2016), our final aim concerns exploring a moderation model (i.e., considering the potential moderating role of reasons on SAp and SAv goals and their relation to performance and emotion among parkrunners). *Our fifth hypothesis for this research expects more self-determined (autonomous) reasons to moderate the relationship between SAp goals to performance and pride via challenge appraisals (H^5a^). It was also tentatively hypothesized that less self-determined (controlled) reasons could moderate the indirect relationship between SAv goals toward performance and shame via threat appraisals (H^5b^)*.

### The present research

For the first time in the sport-based AGA-SDT literature, the present study adopted a longitudinal prospective design to exclusively focus on self-based goals (approach and avoidance) as they have been previously ranked the most important goal for athletes participating in a running event (e.g., Krouse et al., 2011; Delrue et al., 2016; Roebuck et al., 2018). By utilizing the unique approach of a conditional process model within this achievement context, the present researchers examined the potential moderating role of self-determined reasons in explaining the indirect relationship of self-based goals predicting performance and emotions via stress appraisals. Additionally, in addressing previous design limitations, we targeted a shorter distance event, namely a 5 km parkrun (as prior literature reviewed tended to focus on long-distance runners) whilst sampling a wide-ranging ability of participants (aligned with the population parkrun attracts). To extend upon the above, we hypothesized a series of relationships between goals and their reasons in predicting performance and well-being. We sought to conduct an in-depth exploration of goal complexes (i.e., the interaction of goals and their reasons) in predicting stress appraisals, performance, and discrete emotions. Within this, we were interested in gaining a greater understanding of the processes that may occur through cognitive appraisals of stress (i.e., challenge and threat), for which achievement motivation has been widely empirically supported to play a key influencing role (e.g., Jones et al., 2009). Our hypothesized pathways are depicted in Figure 1.

## Methods

### Participants

Three hundred and twenty-four individuals (*M*_age_ = 45.40; SD = 10.79; 66% females) completed the study, with participation in 203 parkrun events across the UK represented by our final sample. It was a requirement that all participants entering into the study had completed at least one parkrun previously. On average, participants reported running three times per week (*M* = 3.09; SD = 0.63), being coached (*M* = 1.96; SD = 0.20) and affiliated with a club (*M* = 1.68; SD = 0.47) for nearly 2 years.

### Design and procedures

Following institutional and parkrun ethical approval, the current online study (utilizing Qualtrics survey software), adopting a longitudinal, prospective design, was advertised on parkrun UK’s social media platforms. Interested participants were directed to online participant information detailing the purpose and requirements of the study. After gaining digital consent, participants were prompted to and completed a series of short online questionnaires in the lead-up to and shortly following their next targeted parkrun. At Time 1 (T1; 7 days pre-parkrun), self-based goals and their underlying reasons for adopting these goal pursuits were measured. At Time 2 (T2; 24 h pre-parkrun), challenge and threat appraisals of the parkrun were assessed. Finally, at Time 3 (T3; immediately post-parkrun), a measure of objective performance was recorded, along with self-reported pride and shame. Complete data across the three time points were obtained and analyzed for 324 participants (i.e., 77% retention rate). The entire questionnaire (across all three timepoints) took approximately 20 min to fill-out.

### Measures

#### Self-based goal pursuits

Two modified subscales from the 3 × 2 Achievement Goal Questionnaire for Sport (AGQ-S; Mascaret et al., 2015) were employed to capture the strength of participants’ SAp (3 items, e.g., “*to perform better than I have done previously*”) and SAv (3 items, e.g., “*to avoid doing worse than I normally do in this event*”) goals 7 days prior to their next parkrun. Participants responded along a 7-point Likert-scale ranging from 1 (“Strongly disagree”) to 7 (“Strongly agree”). Past sport research has found these subscales yielded excellent internal reliability (e.g., Mascaret et al., 2015), factorial and predictive validity (e.g., Mascaret et al., 2015; Wang et al., 2017), and measurement invariance across age and gender (Wang et al., 2017).

#### Reasons underlying self-based goal pursuits

To measure the reasons underlying self-based goals, we followed a similar procedure used by past research (e.g., Vansteenkiste et al., 2010a,b, 2014a,b). Immediately after participants responded to each goal item, they were asked to identify why they pursued SAp and SAv goals capturing: (1) intrinsic reasons (1 item; e.g., “*Because of the fun and enjoyment it provides me*”), (2) identified reasons (1 item: “*Because I really believe it is an important goal to have*”), (3) introjected reasons (1 item: “*Because I would feel ashamed and guilty if I did not*”), and (4) external reasons (1 item: “*Because others expect me to*”). Individuals responded to items along a 7-point Likert-scale ranging from 1 (“Strongly disagree”) to 7 (“Strongly agree”). This short version measure of goal regulations has demonstrated acceptable reliability and structural validity in sport (Delrue et al., 2016).

Consistent with other SDT-based-studies (e.g., Lutz et al., 2003; Gillet et al., 2010), we used the Relative Autonomy Index (RAI) to reduce the complexity of our model. In the current study, a Relative Autonomy Index (RAI) was calculated twice to reflect the measurement of the reasons underlying each self-based goal. The RAI’s were computed by assigning a weight to each of the motivation subscales depending on their placement along the self-determination continuum (external regulation, −2; introjection, −1; identification, +1; and intrinsic motivation, +2) and then summing these weighted scores. Higher scores reflected more self-determined reasons underpinning the pursuit of a SAp and SAv goal.

#### Cognitive appraisals of stress

An adapted 8-item version of the challenge and threat construal measure (McGregor and Elliot, 2002) was employed to assess participants’ appraisal of their 5K parkrun 24 h pre-event. Individuals responded to the stem “How do you feel about completing tomorrow’s 5K parkrun?” along a 7-point Likert-scale ranging from 1 (“Not at all true of me”) to 7 (“Very true of me”). Sample items from the challenge and threat measure were “*I view this parkrun as a positive challenge*” and “*I view performing this parkrun as a threat*.” The challenge and threat construal measure has exhibited satisfactory levels of internal consistency, factorial validity and predictive validity in past sport research (e.g., Adie et al., 2008; Kavussanu et al., 2014).

#### Performance outcome

Participants’ 5 km parkrun finishing-time (in minutes) was digitally recorded and used as performance outcome in the current study. Lower 5 km finishing times reflected higher levels of performance.

#### Emotions

The pride and shame subscales of the Achievement Emotions Questionnaire (AEQ; Pekrun et al., 2011), were adapted to the current research context to assess these two types of discrete emotions 24 h post-parkrun. Participants responded along a 7-point Likert-scale (1 = “Not at all true of me”; 7 = “Very true of me”) measuring to what extent they experienced pride (10 items; e.g., “*I was proud of how well I ran the parkrun course*”) and shame (10 items; e.g., “*I felt humiliated*”) retrospectively following their parkrun. These two subscales of the AEQ have previously demonstrated very good psychometric properties (Pekrun et al., 2011).

### Measurement model

The measurement model, step one of Anderson and Gerbing’s (1988) approach, was tested to examine how the observed indicators related to their corresponding latent factors (i.e., SAp and SAv goals; challenge and threat appraisals, pride and shame). It should be noted that the 10 observed items used to assess pride and shame were parceled in order to facilitate a better model fit. Three parcels each were created by averaging stronger with weaker items to load separately onto the respective pride and shame factors (Coffman and MacCallum, 2005). Parceled data can help reduce sources of sampling error and the likelihood of observing correlated residuals or dual loadings preventing, and, thus lowering the risk of Type II error (MacCallum et al., 1999). In total, the measurement model comprised 22 indicators linked to their respective latent factors; self-determined reasons underpinning SAp and SAv goals, and parkrun time were estimated as observed variables.

### Data analysis strategy

Only participants who completed a full dataset across all three timepoints (i.e., 324) were included for analysis in this study. SPSS (version 26) was used to calculate descriptive statistics, internal reliability scores, and zero-order correlations for the study variables (see Table 1). Next, the factor structure of each psychological scale used in the current study was initially tested and supported using confirmatory factor analysis (CFA).^1^ We continued to use SEM analyses (Mplus version 7; Muthén and Muthén, 1998) to test the full hypothesized model as recommended by Anderson and Gerbing (1988). In accordance with Hu and Bentler (1999), the current study applied the following cut-off criteria for a range of fit indices to assess model fit: a non-significant (*p* > 0.05) Chi-square (X^2^) value, a Comparative Fit Index (CFI) close to or above 0.95, and values for the Root Mean Square Error of Approximation (RMSEA) equal to or less than 0.06, respectively. To test the hypothesized mediation pathways, researchers examined indirect effects by interpreting the associated confidence intervals (CIs; MacKinnon, 2008) based on 1,000 bootstrap replications. In order to test moderation, interaction terms were created and tested in Mplus.

**Table 1:** Descriptive statistics, internal reliability and zero-order correlations for the study variables.

Variables	1	2	3	4	5	6	7	8	9
Goals									
SAp	–								
SAv	0.53**	–							
Reasons									
RAI_SAp	0.43**	0.17**	–						
RAI_SAv	0.27**	0.34**	0.73**	–					
Outcomes									
Challenge appraisals	0.33**	0.22**	0.37**	−0.10	–				
Threat appraisals	−0.02	0.07	−0.04	0.26**	−0.29**	–			
Pride	0.18**	0.05	0.26**	0.07	0.37**	0.04	–		
Shame	0.05	0.13*	−0.04	0.10	−0.16**	0.12*	−0.40**	–	
Performance	−0.10	−0.09	−0.15**	0.05	−0.07	0.10	−0.06	0.14*	–
*M*	4.80	4.55	10.05	8.70	5.83	1.57	4.80	1.30	30.27
*SD*	1.27	1.50	4.60	5.00	0.85	0.88	1.23	0.71	6.56
*α*	0.85	0.90	0.93	0.95	0.72	0.72	0.94	0.92	–

SAp, self-approach goal; SAv, self-avoidance goal; RAI_SAp, relative autonomy index underpinning self-approach goals; RAI_SAv, relative autonomy index underpinning self-avoidance goals; M, mean; SD, standard deviation; α, Cronbach’s alpha coefficient. *p < 0.05; **p < 0.01. Goals and Reasons were measured at Time 1 (T1); Challenge and Threat Appraisals were measured at Time 2 (T2); Pride, Shame and Performance were measured at Time 3 (T3).

## Results

### Descriptive statistics, internal reliability and zero order correlations

Table 1 presents the descriptive statistics, internal reliability scores and correlation matrix for the study variables. All data was deemed to be normally distributed with skewness and kurtosis data ranging between +/− 2. On average, participants reported moderately high levels of SAp and SAv goal pursuits, high mean scores for more self-determined reasons underlying self-based goals, challenge appraisals and pride, and low average scores for less self-determined reasons, threat appraisals and shame. Based on the average performance time, participants were deemed to be of recreational standard. All scales used in the study were found to have good-to-excellent internal reliability (α > 0.70).

### The hypothesized model

Examination of the full hypothesized model revealed it to be an excellent fit for the data, *X*^2^ (197) = 393.25; *p* < 0.001; CFI = 0.96; RMSEA = 0.05 (90% CI = 0.05–0.06), and explained 17, 8, 4, 24, and 10% of the variance in challenge appraisals, threat appraisals, performance, pride and shame, respectively.

**Figure 2:**
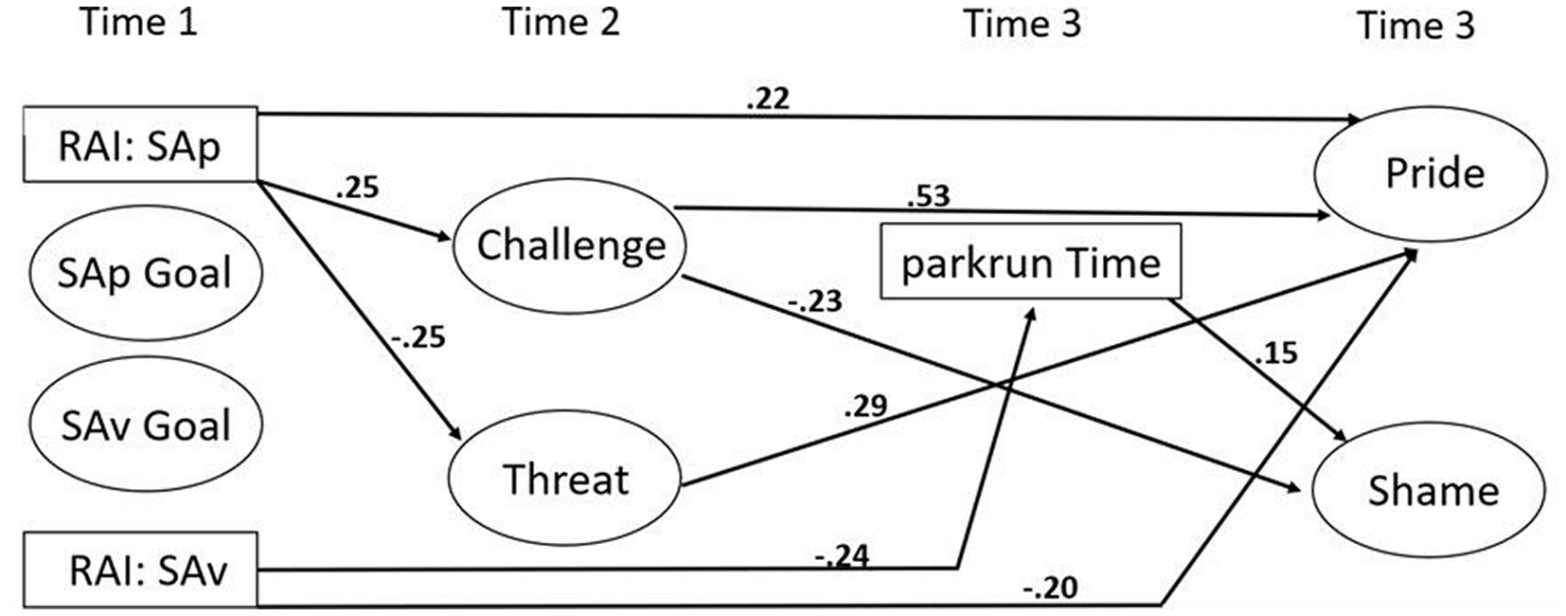
The hypothesized model; significant pathways. SAp = self-approach goal; SAv = self-avoidance goal; RAI_SAp = relative autonomy index underpinning self-approach goals; RAI_SAv = relative autonomy index underpinning self-avoidance goals.

All significant pathways are represented in Figure 2. More specifically, there were significant positive associations between more self-determined reasons underlying SAp goal pursuit with challenge appraisals before, and pride following, a parkrun. In turn, challenge appraisals positively predicted feelings of pride, and were inversely related to shame, respectively. Furthermore, the more self-determined reasons underlying SAp goal pursuit directly and negatively related to threat appraisals. Threat appraisals were also positively associated with pride. The less self-determined reasons underlying SAv goal pursuit was negatively linked with parkrun time and pride. Finally, parkrun time was positively related to shame, albeit weakly.

### Mediational effects

Our final analysis tested a process model by examining the role of cognitive appraisals of stress (i.e., challenge and threat) in the relationship between goal pursuit and their underlying reasons with indicators of performance and discrete emotions (*X^2^*[200] = 490.67; *p* < 0.001; CFI = 0.94; RMSEA = 0.07 [90% CI = 0.06–0.07]). Two indirect, significant pathways emerged indicating the presence of mediation. Firstly, we observed the mediating role of challenge appraisals between the reasons underlying SAp goals and pride (*β* = 0.04; *p* < 0.05). Secondly, we observed the mediating role of threat, also between the reasons underlying SAp goals and pride (*β* = −0.26; *p* < 0.05).

### Moderation effects

Another purpose of the study was to test the potential moderating effects of the reasons underlying goal pursuit with SAp and SAv goals on cognitive appraisals of stress and subsequent performance, pride, and shame. Interaction terms were created for the RAI’s tied directly to their goal pursuit (i.e., RAI_SAp x SAp goals; RAI_SAv x SAv goals) and included in the model. Results suggested a significant moderating effect only of RAI_SAp on the relationship between SAp and threat (*β* = −0.03, *p* < 0.001). However, in this moderation model the unique pathway from SAp to threat became significant and positive (*β* = 0.27, *p* < 0. 01) despite the non-significant correlation (see Table 1) between these two variables (r = −0.02, *p* > 0.05). Hence, the result from the interaction analyses has been considered as a statistical artifact and as such has not been further discussed. Based on the absence of a moderation effect and significant pathways linking the self-based goals to appraisals, performance and emotions, a full process conditional model could not be tested.

## Discussion

Drawing upon the potential integrative possibilities of the AGA and SDT (Vansteenkiste et al., 2014a,b), and extending the work of Delrue et al. (2016), we sought to examine individual’s pursuit of self-based achievement goals and their underlying reasons in influencing the anticipatory stress appraisals, and in turn, performance and emotions of participants in the lead-up to and completion of a parkrun. Furthermore, we were interested in testing the potential moderating role of the reasons underlying self-based goal pursuit on performance, pride and shame as well as the mediating effects of stress appraisals. Our findings demonstrated there is evidence to support the direct effects of more or less autonomous reasons underlying self-based goals, in explaining how parkrunners cognitively appraise a 5 km, and their consequential performance and emotional experiences. There were no moderating effects of the reasons underpinning goal pursuit and no direct goal effects. However, results do reveal the mediating role of cognitive appraisals of stress (via challenge and threat) between underlying reasons of self-based goal pursuits (SAp goals) with pride.

### The hypothesized model

Extending previous research (e.g., Gillet et al., 2017) on the theoretical integration of the AGA and SDT (Vansteenkiste et al., 2014a), the present study sought to examine whether the reasons underlying achievement goal pursuit played any predictive role in our hypothesized sequence of temporal relationships. The findings led us to reject the first set of study hypotheses (H^1a-b^), as achievement goal pursuit revealed no direct effects on any study outcomes (and consequently researchers rejected H^3a-c^, as there were no goal-context interactions observed). These unexpected findings may be explained through the arguments proposed by research and discussed by Deci and Ryan (2000). They suggest when exploring the construct of reasons underpinning achievement goal pursuit, this dimension explains the majority of, if not all motivational processes influencing performance and optimal functioning (via positive emotions in our case), to the point that any potential goal effects that might exist become annulled. Aligned with existing empirical work (see Vansteenkiste et al., 2010a,b; Gaudreau and Braaten, 2016) whereby the influence of underpinning reasons reports more significant effects than the role of achievement goals themselves, our findings among parkrunners seemingly corroborate Deci and Ryan’s (2000) claims.

Rather, all our observed direct effects on study outcomes stemmed from the ‘why’ component of motivation (i.e., the reasons), leading researchers to partially support the second set of hypotheses. Similar to Delrue et al. (2016), we firstly observed autonomous motivation was characterized by an overall positive pattern. That is, in partial support with H^2a^ (and H^4a^), the more self-determined participants were in regulating their SAp goals, the more likely they were to appraise the 5 km parkrun as a challenge and experience pride, and less likely they were to perceive this event to be threatening. The facets underpinning more self-determined (or autonomous) reasons (i.e., volitionally endorsing a goal, placing value upon the outcomes of participation etc.) lend themselves toward satisfaction of the three basic psychological needs (autonomy, competence, and relatedness). Delrue et al. (2016) supported this theoretical proposition in their research and there is also strong evidence existing across other achievement contexts (and grounded in earlier theoretical frameworks), that basic psychological needs satisfaction and frustration influence motivational and emotional outcomes (Bartholomew et al., 2011; Vansteenkiste and Ryan, 2013; Rodrigues et al., 2018; Teixeira et al., 2018). So, it would appear relevant to tentatively propose from our findings that individual’s positive emotional experiences occur as a result of goal pursuit for more self-determined reasons which in turn lends itself to basic psychological needs satisfaction (Deci and Ryan, 2000). However, it is important to clarify, no measure of basic need satisfaction was employed within this study design, so future studies may wish to test this proposition to confirm such relations.

Partially rejecting hypothesis H^2a^, self-determined reasons underlying SAp goals did not reveal any significant relationships with performance (i.e., parkrun time). Perhaps, when considering the nature of parkrun and its promotion as “a run, not a race,” it is plausible that, despite some research recognizing a motive for self-improvement (e.g., Stevinson et al., 2015; Sharman and Nash, 2018; Tulle et al., 2018), individuals actually attribute a greater importance to their running experience and emotions following performing, rather than their actual performance *per se*. This finding is in disagreement with the majority of existing literature who reveal positive links between autonomous motivation and performance (e.g., Vansteenkiste et al., 2014b; Gaudreau and Braaten, 2016) and the results of Delrue et al. (2016). However, it may be further explained through differences in study design and measurement. Firstly Delrue et al. (2016) drew from a population of experienced, competitive runners for whom it has been well-documented that achieving an improved performance is a key requirement for feeling successful (e.g., Roebuck et al., 2018). In contrast, parkrun attracts runners from a variety of backgrounds, including non-competitive, novice runners for whom performance improvement holds importance (as demonstrated through their goal pursuit choices) but possibly, may not be as essential or crucial compared to those regularly competing at a higher standard. Secondly, Delrue et al. (2016) tested autonomous reasons underpinning goal pursuit, whereas in this study, we focused on more or less self-determined reasons representing an account of the level of autonomy participants felt regarding their achievement goal pursuit. Although the RAI measurement method has been utilized in previous research (e.g., Spray and Wang, 2001; Ciani et al., 2011), it does not accurately signify the contributions of SDT’s constructs and so despite influencing discrete emotions, our operationalization of underlying reasons in the current study may have impacted the lack of associations observed with performance.

In partial agreement with H^2b^, we also observed direct relationships between the reason underlying SAv goal pursuit with parkrun time and pride, such that the less self-determined individuals’ reasons were for the pursuit of a SAv goal, the slower they ran and less pride they experienced post parkrun. When considering the characteristics of less self-determined goal striving (e.g., coercion, external rewards and constraints, a lack of values with their goal etc.), it seems theoretically sound to propose that, as a result of the pressure associated with pursuing a SAv goal (with the avoidance focus of this goal naturally providing a negative frame of reference), they ran parkrun in a slower time than their previous attempt (i.e., failing to achieve their goal) and also experienced less pride post-event. Notably, there were no significant relationships between less self-determined reasons for either SAp or SAv goal pursuit and shame within our findings. This finding agrees with results reported by Delrue et al. (2016) who reported similar observations with controlled motivation underpinning self-based goal pursuit. It is possible the detrimental effects expected of less self-determined (or controlled) motivation might be more readily pronounced in a different sporting environment (e.g., a competitive context or within a team sport such as soccer or basketball, where a bad performance may cost a player’s spot on the team). In this situation, failure under pressure has more immediate ramifications, and so may come with a higher personal cost to player’s emotional investment. To extend this explanation, less self-determined motivation in running may have fewer implications on short-term outcomes like (poor) performance and negative emotions (i.e., shame), but rather, might develop over time in the form of dropout. A similar pattern of relationships has been observed in handball (Sarrazin et al., 2002). Dropout is less likely in a parkrun where (1) the distance is shorter, (2) it is accompanied with less mental (and physical) demands on the participant compared to a (ultra)marathon, and (3) they are readily accessible to attempt nationwide every week (Corrion et al., 2018; Quirk et al., 2021). Alternatively, any effects expected or observed from less self-determined motivation may be partly due to the type of achievement goal to which they are tied. Previous literature shows that controlled reasons underlying ‘sub-optimal’ goals (i.e., other-based goals) yield strong negative patterns (Vansteenkiste et al., 2010a,b), while controlled reasons for ‘more adaptive’ goals (i.e., self- or task-based goals) do not carry these negative effects (Vansteenkiste et al., 2014b). Although runners in pursuit of a SAv goal adopted an avoidance focus, which is typically known in sport research to be more negative, its competence referent is related to the self and not in drawing comparison against others, which may cancel out any ill effects such as shame. However, it should also be noted that, comparing competence with one’s own previous performances (i.e., self-based goal) has the potential to elicit ill effects as well, perhaps feelings of disappointment and frustration when not achieving previous standards, for example. Such emotions may demonstrate a direct relationship from the reasons underlying self-based goal pursuit and would provide an interesting avenue for future research.

The present findings also yielded interesting results with respect to cognitive appraisals that warrant discussion. In partial agreement with H^4c^, challenge appraisals yielded significant, positive associations with pride (but not performance), however, this pattern was also observed for threat appraisals. It appears therefore, that irrespective of the fact an individual appraised the task as either a challenge or a threat, they would experience enhanced feelings of pride post-parkrun. The relationship between challenge and pride was expected; if an individual identifies themselves to possess sufficient environmental resources to meet the perceived demands of a task and views the situation as an opportunity for growth or mastery, it has been supported in theory and research, that this challenge appraisal would enhance positive affect (Ntoumanis and Biddle, 1998; Giacobbi et al., 2007) and emotions (Kavussanu et al., 2014). However, observing the same relationships from threat appraisals to pride was unexpected. This may be explained through the fact that we measured discrete emotions retrospectively, after the parkrun had been completed. To that end, it seems reasonable to suggest, that although prior to taking part in the event, parkrunners viewed the activity as threatening, upon successful completion, they could reflect upon their achievement with pride, having effectively overcome doubts regarding their ability to cope with the task. Along these lines, it seems noteworthy to mention that just because an individual perceives a task to be threatening, that does not necessarily undermine the importance they assimilate to their achievement strivings (Lazarus and Folkman, 1984). So, upon attaining their important achievement goal successes, individuals reflect on their performance with pride. Finally, although not explored in this study, it seems plausible to suggest that, despite initially perceiving the parkrun to be threatening, participants employed effective coping strategies throughout their performance which permitted them to eventually experience more positive emotions. Lazarus and Folkman (1984) defined coping as “constantly changing cognitive and behavioral efforts to manage specific external and/or internal demands that are appraised as taxing or exceeding the resources of the person” (p. 141). Research in sport does exist exploring the connections between coping and emotions, with findings highlighting coping could generate adaptive emotions despite facing or operating within stressful situations (e.g., Nicholls et al., 2010). It should be noted, these are tentative interpretations of this finding and as such, requires deeper exploration in future research.

Furthermore, rejecting H^4d^, we observed no significant associations between threat to performance or shame. This was surprising given the links demonstrated in previous literature between threat appraisals and sub-optimal functioning (e.g., Ntoumanis and Biddle, 1998; Giacobbi et al., 2007). Explanations for this may emanate from a measurement and behavioral perspective. Firstly, regarding the measurement of appraisals, this instrument was administered 24 h prior to the parkrun starting. Although recognized as being in relatively close proximity to the event, many things (stemming from personal, environmental, psychological, and emotional adjustments) can change during that time for a participant which ultimately could influence their performance in a more positive manner. Furthermore, changes in cognitive and behavioral efforts during performance related to potentially engaging with effective coping strategies previously discussed, could have superseded any possible negative effects of threat appraisals by readjusting focus on a more positive outlook of possessing an ability to successfully cope with the environmental demands.

A final, interesting result emerged from the findings which was not previously hypothesized. There was a significant, positive association between parkrun time and shame, such that, the higher participants parkrun time (i.e., the slower they ran), the more shame individuals experienced. Despite our findings largely suggesting parkrunners experiences of the event are more directed toward their emotional functioning, rather than their performance, it does appear that when individuals recognize they have not achieved their desired goal (i.e., time), this has a detrimental impact upon their emotions.

### The (mediating) effects of cognitive appraisals

According to Lazarus (1999), cognitive appraisals of a stressful event are proposed to mediate the demands of the objective environment on cognitions, emotions and behavior. Investigating the assumption that achievement goals serve as a perceptual framework for interpreting the objective environment (McGregor and Elliot, 2002), we explored the potential mediational effects between self-based goal pursuit and their underlying reasons to performance and discrete emotions via parkrun appraisals. Specifically, our findings appear to suggest that the more self-determined participants reasons were for SAp goal pursuit (i.e., having a focus on successfully improving previous performances for the pleasure and personal importance it will bring), the more likely they were to experience pride, via viewing their parkrun as a challenge (and as such, having lower threat perceptions). Our findings go beyond existing literature examining the mediating role of cognitive appraisals (e.g., Adie et al., 2008; Kavussanu et al., 2014) by providing evidence for the indirect effects of reasons underlying goals and discrete emotions via appraisals, where previous research has tended to only focus on the achievement goal pursuits.

When considering the mediational findings for SAp goal pursuit, this study demonstrates that it is the regulation underlying this goal, not the goal *per se* that is influential in determining positive, retrospective emotional experiences via higher challenge and lower threat appraisals. As past work has reported, our findings support the supposition that the underlying reasons for goal pursuit explain more variance for studied outcomes above and beyond those relationships from the achievement goal alone (Delrue et al., 2016; Gaudreau and Braaten, 2016; Gillet et al., 2017). Not only do our novel mediational findings further support and extend this work, they open a potential new line of inquiry, identifying an alternative motivational construct influencing challenge and threat states in athletes (i.e., reasons), to those originally proposed in theory [i.e., achievement goals; see Jones et al., 2009 for a summary]. Although our results highlight the positive benefits of more self-determined reasons when endorsing SAp goals and suggest cognitive mechanisms by which these reasons may facilitate a parkrunners emotions, as a novel finding within this context, they should be interpreted with caution in the interim. That is, it is suggested future research is warranted to confirm these relationships before drawing firm conclusions.

### The moderating role of reasons

Rejecting our fifth set of hypotheses (H^5a-b^) the lack of evidence for the potential moderating role of reasons underpinning goal pursuit may be explained in a number of ways. Firstly, by forming a composite RAI score to reflect more or less self-determined reasons for achievement strivings, our study did not exclusively test SDT’s constructs of autonomous and controlling regulations, and therefore, distinct reasons cannot exert any (potential) moderating role. Previous literature (see Gjesdal et al., 2017), although not studying reasons, did differentiate between SDT’s distinct forms of regulation (e.g., intrinsic vs. extrinsic) and observed moderating effects. Further, the scores reflected in the composite RAI variables, did not indicate extremely high pursuit for either more or less self-determined reasons. According to Gsjedal (2017), relatively low levels of self-determined reasons reported may have contributed to the lack of moderating effects. Finally, the absence of moderating findings could be attributed to the fact the main analyses of the present research revealed no direct relationships of achievement goals to any of our studied outcomes.

### Limitations, future directions and practical implications

Despite theoretically advancing previous work (e.g., Delrue et al., 2016), and partially supporting our hypothesized model, our findings have several limitations that should be considered. First, from a conceptual viewpoint, our study focused only on self-based goals (i.e., SAp and SAv). We cannot therefore infer that the same pattern of relationships exists between task- and other-based goals and their underlying reasons on appraisals, emotions and performance. It is difficult from a study design perspective, to fully examine (all six goals from) the most recent conceptualization of achievement goals (i.e., the 3 × 2 AGM; Elliot et al., 2011), especially if additionally considering investigating underlying reasons. However, a fruitful avenue for future research would be to adopt a multi-study approach (i.e., conducting a number of mini-studies within a wider project; see Benita et al., 2014, 2017). Furthermore, when considering the type of achievement goals and the sporting context under investigation, there may be other types of discrete emotions researchers could investigate in the future. In the present study, we focused on outcome-related emotions, however, activity-related emotions could play a key role in these motivation relationships. For example, enjoyment and happiness could be significant indicators of optimal emotional functioning whereas, boredom, anxiety, and anger could prove useful when examining the implications of goal pursuit on emotions among runners and other athletic populations. On a further note, and in relation to pride specifically, our operationalisation of this outcome-based emotion was derived from Pekrun et al. (2006) original taxonomy. However, more recent research has distinguished between different types of pride (i.e., authentic and hubristic, Tracy and Robins, 2007; self- and social comparison-based, Buechner et al., 2018). Thus, future research may choose to examine the relation between self-, task- and other-based goal pursuits on different facets of pride, as well as examining the underlying reasons for these potential effects. Second, the correlational nature of our prospective study design means causality cannot be inferred from the current findings. Future research should consider a cross-lagged panel design, to explore in greater depth, potential recursive relationships between variables with each other over time. Third, in line with study design and according to supporting theory and research (Vansteenkiste et al., 2014a; Gjesdal et al., 2017), we placed both achievement goals and reasons alongside each other when testing our hypothesized and mediation models. However, there is research that exists to suggest that underlying reasons may act as an antecedent for goal pursuit (see Vansteenkiste et al., 2014a). Although we tested such a model (see footnote 2), we did not find any significant relationships. Nevertheless, future research may look to adopt and test this approach in varying sport settings or consider alternative antecedents (e.g., Elliot, 1999). In the context of SDT, the environment (i.e., autonomy-supportive vs. controlling) operating under goal pursuit could be examined as an alternative construct to observe how the social conditions within which one pursues goals, can influence sport how sport performers appraise stress and emotions pre- and post-competition. Fourth, from a measurement perspective, collecting data on participants’ achievement goal pursuit in the present study was time-fixed (T1) as were all additional variables studied (across T2 or T3 only). This did not permit for recording potential fluctuations from participant’s original goal pursuits or psychological experiences either pre- or post-parkrun. Future researchers conducting longitudinal research with several time points of data collection may wish to include this construct and measured variables as time-varying to account for possible change in focus and experiences regarding a sporting event. They could also seek to explore detailed participant insights, perceptions, and emotions regarding their experiences by additionally incorporating a qualitative approach to the research design, such as follow-up focus groups or interviews. Furthermore, extending the study of stress appraisals to include a measure of secondary appraisals (alongside the primary appraisals component the current study focused on) and as such, gaining an understanding of associated athlete coping strategies, may provide a more complete overview of an individual’s (positive and/or negative) stress experience. Associated with this, the present research did not include any objective markers within the design and given the theoretical underpinnings of the revised TCTSA having a Biopsychosocial Model focus, future research could consider exploring biological indicators such as (cortisol and secretory immunoglobulin A [S-IgA]) to advance knowledge on stress response in sport. Fifth, for parsimonious reasons in building our hypothesized model (i.e., in attempting to find the simplest accurate explanation for our investigated psychological constructs), we formulated a RAI, corresponding to individual’s more or less self-determined reasons for self-based goal pursuit. Although this has often been done previously in the SDT-AGA literature (e.g., Spray and Wang, 2001; Ciani et al., 2011) it is important to clarify that this does not represent SDT’s distinct constructs of autonomous and controlling reasons underpinning achievement goals as proposed by theory (e.g., Vansteenkiste et al., 2014a). Therefore, we cannot draw absolute conclusions on the contribution of SDT’s autonomous and controlling reasons underlying goal pursuit toward attaining optimal emotions and performance. Finally, due to the specific population sample we recruited, questions concerning ecological validity and to what extent our findings can be generalized beyond runners could arise. To address this, future research may consider replicating our research design with athletes from alternative sporting contexts.

The current results suggest parkrunners should consider their reasons for engaging with this event, above and beyond what they want to achieve from it (related to their performance [i.e., their achievement goal]). As per common trends in the literature, pursuit of approach-based goals will ensue a range of adaptions, however, the present research suggests engaging with an approach-based parkrun goal for more self-determined reasons, that is for the fun, enjoyment and interest associated with it, will encourage direct relations with a host of well-being outcomes, such as perceiving the event to be more (positively) challenging and in turn, the athlete will experience greater positive emotions (such as pride). Participants in parkrun should avoid taking part in this event for less self-determined reasons (e.g., feeling controlled, guilty, pressurized by an external source) as this is more likely to lead to a poorer parkrun performance (slower time) and consequently, less feelings of pride post-parkrun.

## Conclusion

The present research demonstrates there was evidence to support the value of using SDT as a complimentary framework for AGA in that our findings showed important implications for the regulation of SAp and SAv goals, as opposed to the intensity of pursuing these goals *per se*, in forming anticipatory appraisals and holding subsequent emotional experiences following performing a parkrun. This is consistent with SDT’s theoretical propositions, which posit that if an activity represents the values and interest of the inner self, the achievement process will lead to positive outcomes (Sheldon and Elliot, 1999). Contrary to theoretical propositions (e.g., Vansteenkiste et al., 2014a; Gaudreau and Braaten, 2016) and past empirical work (e.g., Gillet et al., 2014), we failed to support the moderating role of reasons underlying goal adoption on the effects of achievement goals on appraisals, performance, and discrete emotions experienced by parkrunners when considering self-based goals. Taking this into account, further replication of our work is necessary before drawing firm conclusions or practical implications regarding the consequences of integrating these two motivational frameworks within sport.

## Data availability statement

The raw data supporting the conclusions of this article will be made available by the authors, without undue reservation.

## Ethics statement

The studies involving human participants were reviewed and approved by Coventry University and the parkrun Research Board @ Sheffield Hallam University. The patients/participants provided their written informed consent to participate in this study.

## Author contributions

MM: conceptualization, methodology, formal analysis, investigation, writing – original draft, writing – review and editing, and visualization. JA: supervision, conceptualization, methodology, formal analysis, and writing – review and editing. CT: formal analysis, writing – review and editing. All authors contributed to the article and approved the submitted version.

## Funding

The University of Northampton will fully cover the article processing fees, should it be successful in achieving publication status, following the review period. All data underpinning this publication are openly available from the University of Northampton Research Explorer, inquiries regarding the datasets should be directed to the corresponding author MM mairi.mulvenna@northampton.ac.uk.

## Conflict of interest

The authors declare that the research was conducted in the absence of any commercial or financial relationships that could be construed as a potential conflict of interest.

## Publisher’s note

All claims expressed in this article are solely those of the authors and do not necessarily represent those of their affiliated organizations, or those of the publisher, the editors and the reviewers. Any product that may be evaluated in this article, or claim that may be made by its manufacturer, is not guaranteed or endorsed by the publisher.

## Footnotes

^1^The full set of results for the CFAs are available upon request from the first author.
